# The Oncogenic Role of APC/C Activator Protein Cdc20 by an Integrated Pan-Cancer Analysis in Human Tumors

**DOI:** 10.3389/fonc.2021.721797

**Published:** 2021-08-30

**Authors:** Fei Wu, Yang Sun, Jie Chen, Hongyun Li, Kang Yao, Yongjun Liu, Qingyong Liu, Jiaju Lu

**Affiliations:** ^1^Shandong Provincial Key Laboratory of Radiation Oncology, Shandong Cancer Hospital and Institute, Shandong First Medical University and Shandong Academy of Medical Sciences, Jinan, China; ^2^Department of Urology, The First Affiliated Hospital of Shandong First Medical University, Jinan, China; ^3^Department of Dermatology, Qilu Hospital, Cheeloo College of Medicine, Shandong University, Jinan, China; ^4^Department of Urology, Jinan Central Hospital, Cheeloo College of Medicine, Shandong University, Jinan, China; ^5^Department of Urology, Shandong Provincial Hospital, Shandong First Medical University, Jinan, China

**Keywords:** cdc20, pan-cancer, prognosis, ubiquitination, immune

## Abstract

The landscape of *CDC20* gene expression and its biological impacts across different types of cancers remains largely unknown. Here, a pan-cancer analysis was performed to analyze the role of Cdc20 in various human cancers. Our results indicated that the expression levels of the *CDC20* gene were significantly elevated in bladder cancer, breast cancer, colon cancer, rectum cancer, stomach cancer, esophageal cancer, head and neck cancer, kidney cancer, liver cancer, lung cancer, prostate cancer, pancreatic cancer, and uterine cancer. In addition, the expression of *CDC20* was significantly and positively correlated with the increase of clinical stages in multiple cancer types, including breast cancer, kidney cancer, and lung cancer, et al. Among 33 cancer subtypes in the TCGA dataset, the high expression of *CDC20* was correlated with poor prognosis in 10 cancer types. Furthermore, the abundance of phosphorylated Cdc20 in the primary tumor was elevated and correlated with increased tumor grade. Next, we sought to elucidate the oncogenic role by analyzing its association with immune infiltration. For most cancer types, the *CDC20* expression was positively correlated with the infiltration of cancer-associated fibroblasts and myeloid-derived suppressor cells. To further understand its functional activity, we explored the classic Cdc20 downstream substrates, which were found to be mutually exclusive with the expression of Cdc20. Moreover, the pan-cancer analysis of the molecular function of Cdc20 indicated that BUB1, CCNA2, CCNB1, CDK1, MAD2L1, and PLK1 might play a critical role in interaction with Cdc20. The abundance of Cdc20 was further validated at transcriptional and translational levels with a publicly available dataset and clinical tumor tissues. The knockdown of Cdc20 dramatically inhibited tumor growth both *in vivo* and *in vitro*. Therefore, our studies delineated the oncogenic role of *CDC20* and its prognostic significance at the pan-cancer level and proved its functional activity in Cdc20 high expression cancer types. Our studies will merits further molecular assays to understand the potential role of Cdc20 in tumorigenesis and provide the rationale for developing novel therapeutic strategies.

## Introduction

The anaphase-promoting complex/cyclosome (APC/C), which promotes the exit of mitosis has been recognized as one of the major driving forces governing cell cycle regulation in both normal cells and cancer (1). As a multi-subunit E3 ubiquitin ligase, APC/C is involved in the various important cellular process through promoting the ubiquitination and degradation of key cell fate-determining proteins. In doing so, two important adaptor proteins, Cdh1 and Cdc20, are responsible for recognizing the substrate and facilitating the interaction between the substrate factors and the APC/C (2). Both Cdh1 and Cdc20 contain seven WD40 repeats for protein binding, serving as the substrate recognizing subunit of APC, recruiting substrates with the Destruction Box (D-box) motif. Since APC/C^Cdh1^ remains active in the G_1_ phase and late M phase, which controls G_1_/S transition to S and G2 phase, Cdh1 is typically considered to be a tumor suppressor (3). Besides, APC^Cdc20^ exerts its biological functions during the transition from metaphase to anaphase through the destruction of multiple cell cycle regulators. Emerging evidence indicates that APC^Cdc20^ exhibits an oncogenic role by accelerating mitotic progression and multiple cell cycle-independent manners (2). However, the exact role and landscape of *CDC20* gene expression and its biological impacts across different types of cancers remains largely unknown.

Emerging evidence indicated that Cdc20 might possess the oncogenic activity and genetic ablation of *CDC20* significantly inhibits tumorigenesis *in vivo* (4). In addition, depleting *CDC20* in various cancer cell lines led to the blockade of mitotic followed by cell death. These studies suggested that inhibiting *CDC20* might lead to the increase of cell death, which supports the notion that Cdc20 could be a potential anti-cancer therapeutic target. In keeping with these findings, inactivating APC by an IR-motif-mimetic inhibitor, pro-TAME, also induced cell death in a group of cancer types (5, 6). Moreover, Spain, a more specific Cdc20 inhibitor, inhibits the oncogenic function of Cdc20 by directly interfering with the interaction between Cdc20 and its substrates, leading to the blockade of mitotic exit in human cancer cells (7). Furthermore, Cdc20 is highly expressed in multiple types of common human tumors including prostate (8), breast (9), cervical (10), glioblastoma (11), and ovarian tumors (12). Notably, high expression of *CDC20* has been reported to be tightly associated with advanced clinical stages and poor prognosis in human cancers, such as prostate cancer (8). These findings indicate that Cdc20 may be used as a prognostic marker and therapeutic target in human cancers. But the exact cancer types, in which Cdc20 could be potentially targeted, have not been clearly identified.

Cdc20 exerts its biological role largely by targeting its downstream substrate protein for ubiquitination and subsequent degradation in the proteasome pathway. Recently, multiple substrates of Cdc20 have been identified, such as p21, CyclinB, Mcl1, and Bim (6, 13, 14). The primary physiological role of Cdc20 in regulating the cell cycle, while its mutants or inhibition could block the cell cycle progression from anaphase to the next cycle. Therefore, most of the Cdc20 substrates are involved in cell cycle regulation, such as p21 and CyclinB (13, 14). Further investigation found that Cdc20 could regulate apoptosis through targeting Mcl1 or Bim for ubiquitination and degradation (6, 15). Interestingly, it has been reported that Cdc20 also plays a role in ciliary disassembly, catalyzation of Mad2-Cdc20 assembly at unattached kinetochores, and regulation of oxygen homeostasis (16–18). It is probably that the specific substrates of Cdc20 across all types of human cancers remain largely unknown. Therefore, our understanding of how Cdc20 functions in different cancer and its exact biological role in specific cancer types are limited and need to be clarified, which could further guide the molecular exploration and targeted therapy of Cdc20 in cancer research.

In this study, a pan-cancer analysis was performed to analyze the role of Cdc20 in human cancers, including pancreatic cancer, breast cancer, kidney cancer, prostate cancer, colorectal cancer, lung cancer, glioblastomas, bladder, hepatocellular carcinoma, and other cancers, at transcriptomic and proteomic levels. Moreover, mRNA expression, protein abundance, phosphorylation levels, and immune infiltration were integrated and analyzed to further address the biological function and clinical significance of Cdc20 overexpression in various types of cancers, which could merit molecular assays for further exploration of the potential role of Cdc20 and its phosphorylation in tumorigenesis.

## Materials and Methods

### *CDC20* Transcription Analysis

The expression difference of *CDC20* between human cancers and corresponding normal tissues from the TCGA project was analyzed and visualized with the R Version 4.0.3. The clinical information and gene expression RNAseq data of 33 types of cancer (TCGA Pan-Cancer, PANCAN) was downloaded from the UCSC Xena browser (http://xena.ucsc.edu/, accessed by January 20, 2021). The abbreviations of cancer types in TCGA were listed in **Supplementary Table 1**. The ggplot2 package was used to visualize the expression levels of *CDC20*, and the Wilcoxon rank-sum test was used for comparison. For certain tumors with significantly elevated *CDC20* expression in tumor tissues according to the TCGA dataset, GEPIA2 (Gene Expression Profiling Interactive Analysis, http://gepia2.cancer-pku.cn/#index) was applied to verify the difference of mRNA expression. The “Expression analysis-Expression DIY” module of the GEPIA2 webserver was used to calculate and visualize the expression difference between the indicated cancers and the corresponding normal control of the GTEx project(Genotype-Tissue Expression Project, https://www.genome.gov/Funded-Programs-Projects/Genotype-Tissue-Expression-Project). The analyzing parameters were as follows: log2FC (fold change) cutoff =1, *p*-value cutoff = 0.01, log2(TPM + 1) was used for log-scale, Jitter Size = 0.4, and Match TCGA normal and GTEx data were selected. Moreover, the plot of the pathological stage was applied to visualize *CDC20* expression in specific tumors to further understand the association of *CDC20* with progression and prognosis. Log2(TPM + 1) was used for log-scale. To further validate the transcriptional change of *CDC20*, gene expression data of indicated cancers were downloaded from Oncopression Database (http://www.oncopression.com/), followed by analyzation and visualization with GraphPad Prism.

### Survival Analysis

To explore the association of *CDC20* expression with the prognosis of multiple human cancers, we obtain the overall survival (OS) and disease-free survival (DFS) significance map of *CDC20* according to TCGA datasets by the “Survival Map” module of GEPIA2. The analyzing parameters were as follows: FDR was used for p-value adjustment; the significance level was 0.05; survival time units were months. The grouping method was based on the mRNA expressing level of *CDC20*, in which the median value was set as the cutoff thresholds for the high *CDC20* group and low *CDC20* group. The hazard ratio was calculated based on the Cox PH model. The 95% confidence interval (CI) was added as the dotted line.

### The Abundance of Total Protein and Phosphorylation

Next, we sought to confirm the consistency of *CDC20* expression between the transcriptome and the proteomics data from TCGA and CPTAC(Clinical Proteomic Tumor Analysis Consortium). To this end, the protein expression data of multiple types of tumors from CPTAC(colon cancer, breast cancer, ovarian cancer, clear cell renal cell carcinoma, and uterine corpus endometrial carcinoma) were statistically analyzed and visualized by UALCAN (http://ualcan.path.uab.edu/analysis-prot.html) (19). In this study, we explored the protein abundance of the Cdc20 total protein or phosphorylated Cdc20 (with phosphorylation at S41 and T106 sites) between the selected cancer and corresponding normal tissues.

### Immune Infiltration Analysis

*CDC20* has been reported to be correlated with the immune infiltration of CD8^+^T cells, monocytes, and exhausted T cells. However, the association between *CDC20* and cancer-associated fibroblasts(CAFs) has not been explored (20). To this end, the “Immune-Gene” module of the TIMER2.0 (http://timer.cistrome.org/) was applied to analyze and visualize the interaction between *CDC20* and cancer-associated fibroblasts (CAFs), as well as myeloid-derived suppressor cells (MDSCs) in TCGA datasets (21). The p-values and correlation values were calculated by the purity-adjusted Spearman’s rank correlation test. The results were visualized as a heatmap by Spearmans’ Rho value, as well as a scatter plot. Multiple algorithms, including TIMER, XCELL, MCPCOUNTER, and EPIC, were applied for immune infiltration estimations, and the infiltration level of MCPCOUNTER and EPIC were visualized.

### *CDC20*-Related Gene Enrichment Analysis

To further explore the role of *CDC20* in biological functions across different types of cancers, the Cdc20 interaction protein and the transcriptional correlated genes of *CDC20* were obtained and synthetically analyzed. In brief, the “Similar Gene Detection” module of GEPIA2 (http://gepia2.cancer-pku.cn/#similar) was used to analyzed the top 100 significantly correlated genes of *CDC20* based on the 33 TCGA tumors and TCGA normal tissues. Moreover, STRING (https://string-db.org/) was used to generate the Cdc20 interacting protein list based on the experiments from updated literature. Next, we used the “Gene_Corr” module of TIMER2.0 to obtain a list of correlated genes and visualize the data with heatmap by Prism 8, according to the partial correlation (cor) and P-value in the purity-adjusted Spearman’s rank correlation test. In addition, the pan-cancer data from TCGA were downloaded to local from http://xena.ucsc.edu/ and analyzed by R language software (R 4.0.3, https://www.r-project.org/). A pairwise gene Pearson correlation analysis of *CDC20* and targeted genes were performed by R using the ggplot2 package. The *p*-value and the correlation coefficient were indicated in figure pannels. The intersection analysis of Cdc20 interacting genes and proteins was visualized by Venny2.1 (https://bioinfogp.cnb.csic.es/tools/venny/). We also performed gene ontology analysis (https://david.ncifcrf.gov/) and KEGG (Kyoto encyclopedia of genes and genomes) pathway analysis with the Cdc20 correlated genes.

### Cell Lines

All cell lines were purchased from American Type Culture Collection and tested for mycoplasma contamination. HEK293T, A498, Caki-1, PC3, VCaP, C4-2B, A549, and HBE cells were maintained in Dulbecco’s Modified Eagle’s Medium (DMEM) containing 10% fetal bovine serum (FBS), 100 unit/ml penicillin and 100 mg/ml streptomycin. 786-O, 769P, LNCaP, 22Rv1, TRAMP-C2, NCI-H1299, NCI-H460, NCI-H1975, and PC-9 cells were cultured in PRMI-1640 medium. ACHN cells were cultured in Minimum Essential Media (MEM) with 10% FBS.

### Lentiviral Packaging and Infection

To generate stable cells, lentiviral shRNA virus packaging and subsequent infection of various cell lines were performed according to the protocol described previously (4). Briefly, lentiviral constructs were co-transfected with the pCMV-dR8.91 (Delta 8.9) plasmid containing gag, pol, and rev genes, and the VSV-G envelope-expressing plasmid into HEK293T cells. Transfection with Lipofectamine (Invitrogen) was performed according to the manufacturer’s instructions. After transfection 48 h and 72 h, the supernatant was harvested and filtered through a 0.45 μm syringe filter and used to infect cells in the presence of 4 mg/ml Polybrene. Infected cells were selected using puromycin (2 mg/ml) for three days.

### qRT-PCR Analyses

Total RNA was extracted using the RNeasy mini kit (QIAGEN), and the reverse transcription reaction was performed using Power SYBR Green PCR Master Mix (ABI, 4367659). The real-time RT-PCR was performed with the 7500 Fast Real-time PCR system (ABI). Primers were listed as follows: human *CDC20*: Foward primer, GCACAGTTCGCGTTCGAGA, Reverse Primer CTGGATTTGCCAGGAGTTCGG; mouse *CDC20*: Forward Primer, GTTCGTGTTCGAGAGCGATTT, Reverse Primer, CTAGGGGTGGTCTGAACCTT.

### Immunoblot

Cells were lysed in RIPA lysis buffer (Beyotime, China) supplemented with protease inhibitors (Complete Mini, Roche) and PMSF (Sigma, Germany). The cell lysates were centrifuged at 12,000 g for 10 min at 4°C. Protein concentrations were measured using a Bal using the Bradford Protein Assay Kit (Beyotime, China). Equal amounts of protein were resolved by SDS–PAGE using a standard protocol as previously reported (4). Briefly, total cellular proteins separated with the 10% SDS-PAGE gel were transferred to a polyvinylidene fluoride membrane (Millipore, USA). The PVDF membrane was then incubated with antibodies overnight at 4°C. The proteins were visualized with ECL Western Blotting Substrate (Millipore, USA). The antibodies that were used in this study were as follows: mouse monoclonal antibody against Cleaved Parp1 (sc-56196, Santa Cruz, USA), Vinculin (ab130007, Abcam, USA); rabbit polyclonal antibody against Caspase-3 (9662, Cell Signaling Technology, USA), Cleaved Caspase-3 (9664, Cell Signaling Technology, USA), Cdc20 (10252-1-AP, Proteintech, USA), Actin (AF5003, Beyotime, China).

### Colony Formation

Equal number of control cells or cells with *CDC20* knockdown were plated in triplicate and grown in 6-well plates. After 2 weeks, cells were washed with PBS buffer solution before fixation using methanol for 30 min. Crystal violet staining solution was used to dye cells. Colonies consisting of a nonoverlapping group of at least 50 cells were counted manually by a blinded reader using a light microscope.

### Annexin-V/PI Staining

For detection and quantification of apoptosis, cells were treated with TNF-α (20 ng/ml) and Cycloheximide (10μM) for 4 hours before staining. Then cells were harvested and co-stained with FITC Annexin V and Propidium Iodide (FITC Annexin V Apoptosis Detection Kit I, BD Bioscience) according to the manufacturer’s instructions. Stained cells were detected with a BD FACSCalibur System (BD) at the Shandong First Medical University.

### Animal Experiments

C57BL/6J mice were obtained from Beijing HFK Bioscience Co.LTD. All mouse experiments were conducted under a protocol approved by the Animal Care and Use Committee of *Shandong First Medical University and Shandong Academy of Medical Sciences*. For TRAMP-C2 subcutaneous tumor models, 6 weeks old male C57BL/6J mice were allocated randomly into two groups (five mice per group). All experiments were conducted under aseptic conditions. Control shRNA or *CDC20* shRNA infected TRAMP-C2 cells (5 ×10^5^) suspended in 0.1 mL serum‐free culture medium was injected into the upper flank region. All tumors were measured by a blinded reader using Vernier calipers every 3 days. The formula used to calculate tumor volumes was as follows: volume(mm^3^)=length(mm)×width(mm)^2^/2. Four weeks later, mice were sacrificed by cervical dislocation and the tumors were resected and photographed for further analysis. All mice were housed in a pathogen-free environment with free access to food and water at the animals’ facility of *Shandong First Medical University* and were handled in strict accordance with the ‘Guide for the Care and Use of Laboratory Animals.

### Clinical Tumor Samples

Fresh specimens and matching adjacent normal renal tissues were obtained from kidney cancer patients who had undergone radical nephrectomy at the Urology Department, The First Affiliated Hospital of Shandong First Medical University (Jinan, Shandong Province, China) between January 2019 and May 2020. The patients had not received any local or systemic therapeutics before surgery. Normal tissues were sampled at least 2 cm away from the tumor margin. All subjects were ethnic Han Chinese. This study was approved by the institutional review boards of Shandong First Medical University. Written informed consent was obtained from each subject. All experimental methods were in accordance with the Helsinki Declaration.

### Statistical Analyses

All images are representative of results from three independent experiments unless otherwise stated. Statistical analyses were performed with Excel (Microsoft) or R Version 4.0.3 software. The unpaired Student’s t-test was used for comparisons in experiments with only two groups. In experiments with more than two comparison groups, ANOVA was performed followed by Fisher’s least significant difference or the Bonferroni test for pairwise comparisons among three and greater than three groups, respectively. Error bars represent standard deviation. The statistical significance computed by the Wilcoxon rank-sum test or t-test is annotated by the number of stars (*: P < 0.05, **: P < 0.01, ***: P < 0.001).

## Results

### *CDC20* Gene Expression in the Pan-Cancer Analysis

**Figure 1:**
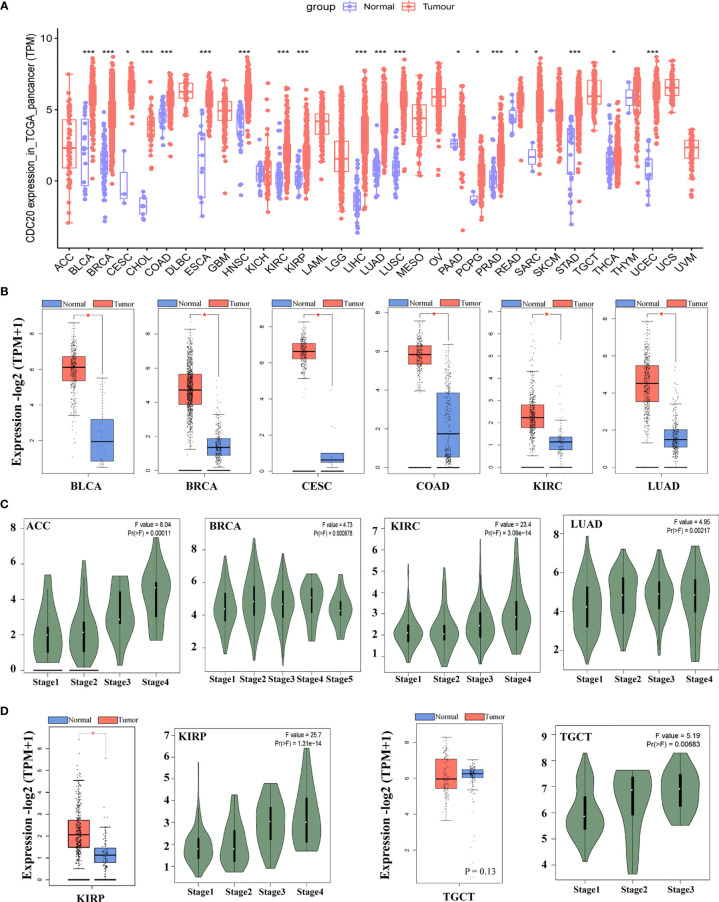
The mRNA expression of *CDC20* gene in different types of TCGA tumors and different pathological stages. **(A)** The mRNA expression levels of the *CDC20* gene in different types of cancers and corresponding normal tissues or specific cancer subtypes from TCGA were statistically analyzed by R version 4.0.3. *P < 0.05; ***P < 0.001. **(B)** Box-plots of mRNA expression for representative cancers (BLCA, BRCA, CESC, COAD, KIRC, LUAD) with significantly elevated *CDC20* expression in malignant tissues according to the TCGA pan-cancer dataset. Besides, expression data of normal tissues from GTEx were included as normal controls. **(C)** The mRNA levels of the *CDC20* gene were analyzed by clinical stages (stage 1, stage 2, stage 3, and stage 4) of ACC, BRCA, KIRC, and LUAD. Log_2_ (TPM+1) was used for log-scale. **(D)** The mRNA levels of the *CDC20* gene were analyzed by clinical stages (stage 1, stage 2, stage 3, and stage 4) of KIRP and TGCT. Log_2_ (TPM+1) was used for log-scale.

Firstly, we explore the mRNA expression pattern of the *CDC20* gene in various cancer and normal tissues of 33 cancer types or subtypes from the TCGA database using the TIMER 2.0 approach and R, respectively. The expression levels of the *CDC20* gene were significantly elevated in the cancer tissues of BLCA, BRCA, CESC, CHOL, COAD, ESCA, HNSC, KIRC, KIRP, LIHC, LUAD, LUSC, PAAD, PCPG, PRAD, READ, STAD, THCA, UCEC, compared with corresponding normal tissues (**Figure 1A** and **Supplementary Table 1**). In addition, for several cancer types, including ACC, DLBC, LAML, MESO, OV, UCS, UVS and SARSonly the transcriptome data from tumor tissues were obtained in TCGA, for which the analysis with normal tissues was not available (**Figure 1A**). To this end, *CDC20* gene expression was increased in most cancer types of homo sapiens, it is interesting to further explore the oncogenic role of *CDC20* at the pan-cancer level. Next, to verify the overexpression pattern of *CDC20* in indicated cancer types, the GTEx dataset was included and served as control groups with the TCGA normal datasets. As a result, the expression difference of *CDC20* between tumor tissues and control groups were significantly increased in BLCA, BRCA, CESC, CHOL, COAD, ESCA, HNSC, KIRC, KIRP, LIHC, LUAD, LUSC, PAAD, PRAD, READ, STAD, UCEC, which was consistent with the analyzing of TCGA dataset alone (**Figures 1B** and **S1A–J**). For the analysis of ACC with the GTEx dataset included, which has no normal control in TCGA, the expression of *CDC20* was also significantly elevated. However, for TGCT, the *CDC20* expression levels failed to show the significant change between tumor tissues and control groups (**Figure 1D**, P>0.05). As a result, the overexpression of the *CDC20* gene in malignant tissues could be a general biological alternation in the tumor, which further supports the oncogenic role of *CDC20*.

Next, we sought to understand the role of *CDC20* in prognosis by analyzing its correlation with clinical staging. The “Pathological Stage Plot” module of GEPIA2 was used to observe the correlation between *CDC20* mRNA levels. Firstly, the cancer types with significantly elevated *CDC20* gene in the previous analysis were selected, and the expression of *CDC20* was significantly and positively correlated with the increase of clinical stages in indicated cancers types, including ACC, BRCA, KIRC, LUAD, (**Figure 1C**, P<0.05). Furthermore, we wondered whether the expression of *CDC20* could be dramatically increased in the late stage of cancer, but not the early stage. To be more specific, we hypothesized that the increased expression of *CDC20* in the high pathological stage could be diluted by the normal expression of *CDC20* in the early stage of cancers. To this end, we observed that the expression of *CDC20* was significantly increased in stage 3 and stage 4 of KIRP, and stage2 and stage 3 of TGCT, but not the earlier stages in the presented cancer types (**Figure 1D**, P<0.05). Together, these results demonstrate that the expression of *CDC20* significantly increased and correlated with increased clinical stages in multiple cancer types.

### Survival Analysis of *CDC20*

**Figure 2:**
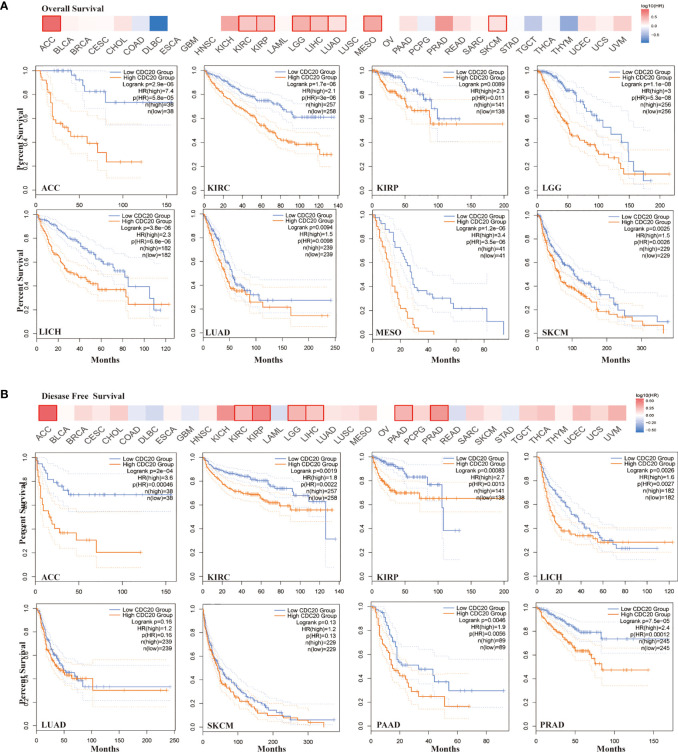
Correlation between *CDC20* expression levels and survival of different cancers from TCGA dataset. **(A)** The heatmap of overall survival analysis across 33 types of cancer from TCGA dataset (upper). In the heatmap, the bold box border indicated that the overall survival difference between CDC20 high and low expression cases was significant (P < 0.05). The survival plot of indicated cancer types was presented below the heatmap. **(B)** The heatmap of disease-free survival analysis across 33 types of cancer from TCGA dataset (upper). In the heatmap, the bold box border indicated that the disease-free survival difference between CDC20 high and low expression cases was significant (P < 0.05). The survival plot of indicated cancer types was presented below the heatmap.

To understand the role of *CDC20* expression in the prognosis of different cancer types, the cases were grouped into low-*CDC20*-group and high-*CDC20*-group according to the expression median of *CDC20* mRNA. Next, we investigated the correlation of *CDC20* mRNA expression with the prognosis of patients with different cancer types in TCGA. As shown in **Figure 2A**, the high expression of *CDC20* was negatively correlated with the overall survival (OS) in multiple cancer types, especially ACC, KIRC, KIRP, LGG, LIHC, LUAD, MESO, and SKCM. In ACC, the hazard ratio (HR) was the highest (HR=7.4, P<0.001) among the 8 significantly correlated cancer types from 33 cancer types in TCGA (**Figure 2A**). Moreover, the high expression of *CDC20* was negatively correlated with the disease-free survival (DFS) in multiple cancer types, especially ACC, KIRC, KIRP, LGG, LIHC, PAAD, and PRAD (**Figure 2B**). Similarly, the HR of DFS was the highest (HR=3.6, P<0.001) in ACC among the 7 significantly correlated cancer types from 33 cancer types in TCGA. For LUAD, MESO, and SKCM, CDC20 expression significantly correlated with the OS, but not PFS. And the *CDC20* expression significantly correlated with the PFS, but not OS, in PAAD and PRAD. Overall, among 33 cancer types in the TCGA dataset, the high expression of *CDC20* was correlated with poor prognosis in 10 cancer types, which further supports its oncogenic role in tumor progression.

### Analysis of Cdc20 Total Protein and Phosphorylation

In this study, we explored whether the transcriptome data is consistent with the transcriptional abundance by the CPTAC dataset, which analyzes the samples from TCGA by reverse-phase protein array (RPPA).

**Figure 3:**
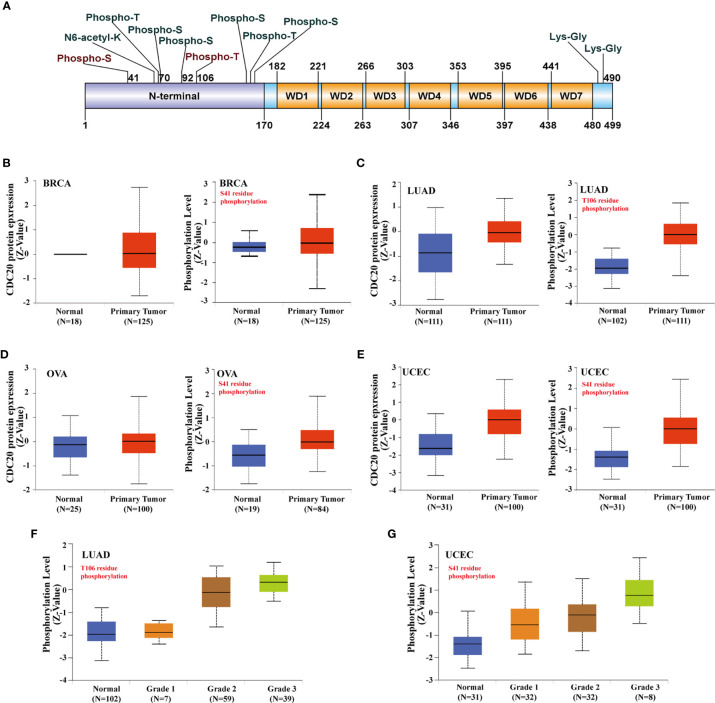
Phosphorylation of Cdc20 protein in different cancer types and its clinical significance. **(A)** The phosphorylation sites of Cdc20 that have been verified are displayed in the schematic diagram of Cdc20 protein. Based on the CPTAC dataset, we analyzed the expression level of total Cdc20 protein and phosphorylated Cdc20 protein (S41 and T106 sites) between primary tumors of selected tumors and corresponding normal tissue *via* the UALCAN. **(B)** The protein abundance of Cdc20 total protein(left) and S41-phosphorylated protein(right) in BRCA tumor tissues and normal tissues, visualized in box plots. **(C)** The protein abundance of Cdc20 total protein(left) and T106-phosphorylated protein(right) in LUAD tumor tissues and normal tissues, visualized in box plots. **(D)** The protein abundance of Cdc20 total protein(left) and S41-phosphorylated protein(right) in OVA tumor tissues and normal tissues, visualized in box plots. **(E)** The protein abundance of Cdc20 S41-phosphorylated protein(right) in UCEC tumor tissues and normal tissues, visualized in box plots. **(F)** The protein abundance of Cdc20 T106-phosphorylated protein(right) in LUAD normal tissues and tumor tissues of different grades (grade1, grade2, grade3), visualized in box plots. **(G)** The protein abundance of Cdc20 S41-phosphorylated protein(right) in UCEC normal tissues and tumor tissues of different grade(grade1, grade2, grade3), visulized in box plots.

As shown in **Figure 3A**, the Cdc20 protein is consist of an N terminal and 7 repeated WD domain. In the N terminal of Cdc20, multiple phosphorylation sites have been verified and functionally annotated. Specifically, phosphorylation at S41 and T106 were frequently reported in the literature, but its role in cancer has not been fully understood. The abundance of total Cdc20 protein was significantly elevated in BRCA, LUAD, UCEC, in which the mRNA expression of *CDC20* was also increased in cancer tissues (**Figures 1A** and **3B**). Moreover, the abundance of total Cdc20 protein was significantly altered in OVA, which was also consistent with previous transcriptomic findings. Next, we sought to study whether the abundance of phosphorylated Cdc20 was consistent with the total Cdc20. Interestingly, the expression levels of phosphorylated Cdc20 in the primary tumor were predominantly increased in primary tissue, with a higher fold change compared with the Cdc20 total protein (**Figures 3B–E**). Importantly, the abundance of S41 phosphorylated Cdc20 was significantly increased in primary OVA tumor tissues compared with normal tissues, while there was no significant difference between tumor and normal in total protein (**Figure 3D**). To be more specific, we explore the prognosis role of phosphorylated Cdc20 by analyzing its abundance in primary tumors of different pathological grades. As shown in **Figure 3F**, we observed that the abundance of T106 phosphorylated Cdc20was significantly increased in grade 2 and grade 3 of LUAD, but not the grade 1 tumor or normal tissues. In UCEC, the abundance of S41 phosphorylated Cdc20 was positively correlated with the increase of tumor pathological grade (**Figure 3G**). Based on this evidence, it is indicated that the phosphorylated Cdc20 could be more functional active in performing its oncogenic role in cancer.

### Analysis of *CDC20* and Immune Infiltration

**Figure 4:**
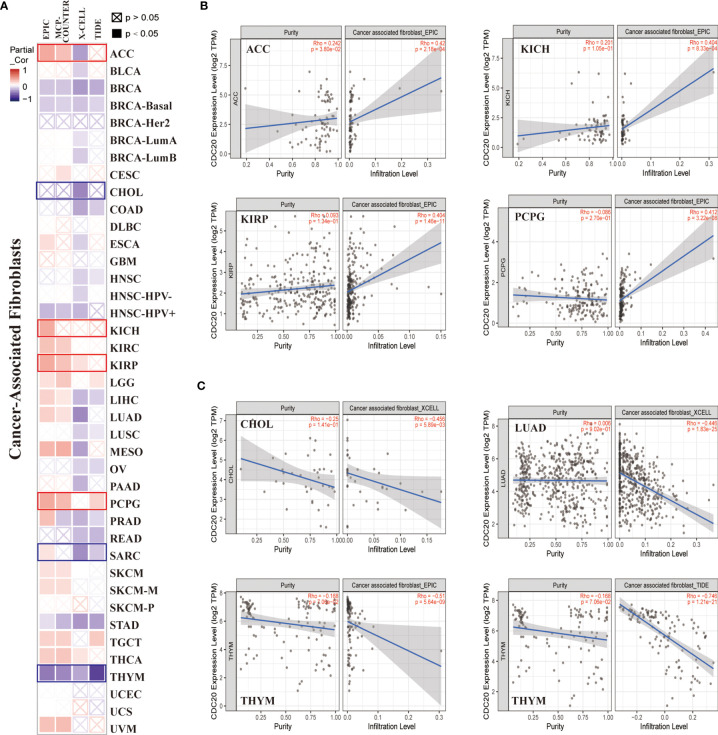
The correlation between *CDC20* expression and immune infiltration of cancer-associated fibroblasts. **(A)** The potential correlation between the expression level of the *CDC20* gene and the infiltration level of cancer-associated fibroblasts across 33 subtypes of cancer in TCGA using different algorithms by TIMER2.0, visualized in a heatmap by Spearmans’ Rho value. **(B, C)** The expression levels of the *CDC20* gene are positively or negatively correlated with the infiltration level of cancer-associated fibroblasts by the cut-off value of 0.4 of Spearmans’ Rho Value in selected cancer types.

Recent studies have reported that *CDC20* was correlated with the immune infiltration of CD8^+^T cells, monocytes, and exhausted T cells. However, the association between *CDC20* and CAFs and MDSCs remains elusive. CAFs in the stroma of the tumor microenvironment were extendedly researched recently and were believed to regulate the function of tumor-infiltrating immune cells including CD8^+^T cells and monocytes. To this end, the “Immune -Gene” module of the TIMER2.0 was applied to analyze and visualize the interaction between *CDC20* and CAFs as well as MDSCs in TCGA datasets, using TIDE, XCELL, MCPCOUNTER, and EPIC algorithms. As shown in the heatmap of correlation, we observed a statistically positive correlation of *CDC20* gene expression and the estimated infiltration value of CAFs among the TCGA tumors of ACC, ESCA, KICH, KIRC, KIRP, LGG, LIHC, LUAD, MESO, PCPG, SKCM, TGCT, but noted a negative correlation for BRCA, HNSC, STAD, and THYM (**Figure 4A**). When the cut-off value of Spearmans’ Rho value was set to 0.4, the scatter plot data of the filtered tumors were presented in **Figures 4B–C**. For example, the *CDC20* expression level in ACC was positively correlated with the infiltration level of CAFs (**Figure 4B**, Rho=0.42, P=2.18e-4) based on the EPIC algorithm. More importantly, the *CDC20* mRNA levels were dramatically and positively correlated with the infiltration of MDSCs, which indicated the immune suppression role of *CDC20* in cancer patients (**Figure S2**). Above all, in most cancer types, the scatterplots were positively correlated, while the correlation was significantly negative in BRCA, as shown in the heatmap, based on all or most algorithms.

### Cdc20 Total Protein and Its Substrates

**Figure 5:**
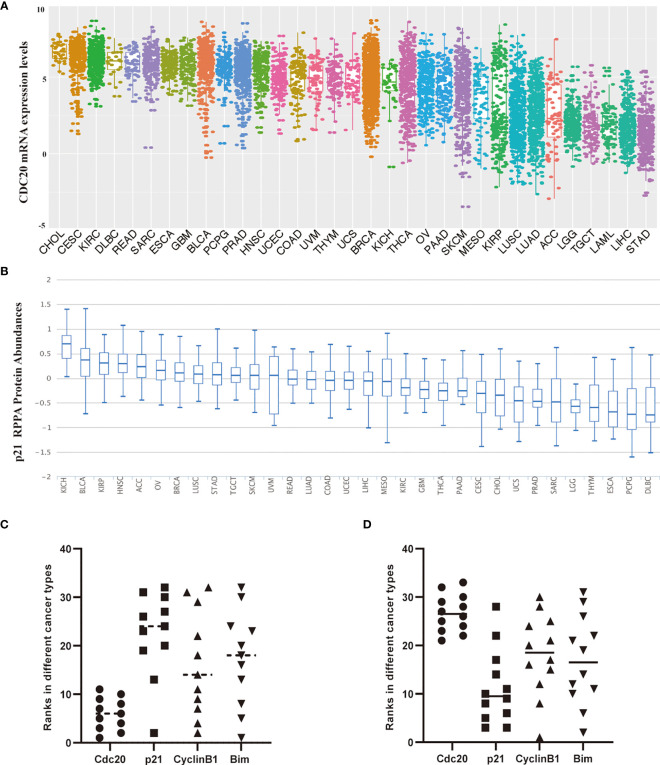
The correlation of protein abundance between Cdc20 total protein and its ubiquitination substrates in different cancer types. **(A)** The mRNA expression levels of the *CDC20* gene in specific cancers or cancer subtypes from the TCGA database. The names of cancer types or subtypes are arranged in descending order of *CDC20* express levels. **(B)** The p21 protein levels in different types of cancers by RPPA using the samples from TCGA. The names of cancer types or subtypes are arranged in descending order of p21 protein abundance. **(C)** Scatter plots visualize the ranks of Cdc20 and its substrates(p21, CyclinB1, and Bim) in specific cancer types. The cancer types in the top 11 ranks with Cdc20 high expression are selected. **(D)** Scatter plots visualize the ranks of Cdc20 and its substrates(p21, CyclinB1, and Bim) in specific cancer types. The cancer types in the last 12 ranks with the Cdc20 expression list are selected.

APC complex exerts its E3 ubiquitin ligase activity largely through recruiting one of several adaptors, such as Cdc20 and Cdh1. Cdc20 recognizes its ubiquitin substrate largely through its N-terminal conserved C-box. The binding between Cdc20 and its substrates promotes ubiquitination and degradation through the proteasome pathway. Herein, to further explore the functional activity in cancer biology, we analyze the abundance of Cdc20 and its substrates at the pan-cancer level. Firstly, we generate a ranking list of cancer types in order of *CDC20* gene expression (**Figure 5A**). Next, three Cdc20 substrates with experimental evidence were included in the analysis. For each substrate, a ranking list of cancer types in order of the protein abundance of the substrate was generated, including p21, cyclinB1, and Bim (**Figure 5A**). If the Cdc20 was functionally active in specific cancer, the protein abundance of its classic substrates should be degraded significantly and down-regulated in RPPA analysis. Therefore, scatter plots were applied to visualize the ranks of Cdc20 and its substrates(p21, CyclinB1, and Bim) in specific cancer types. The cancer types in the top 11 ranks with Cdc20 high expression are selected in **Figure 5C**. The cancer types in the last 12 ranks with the Cdc20 expression list are selected in **Figure 5D**. In keeping with our hypothesis, the ranks of *CDC20* and its substrates tend to be negatively correlated in the scatter plot. Together, these results indicated that Cdc20 is functionally active in the *CDC20* high-expression cancer types.

### CDC20-Related Gene Enrichment Analysis

**Figure 6:**
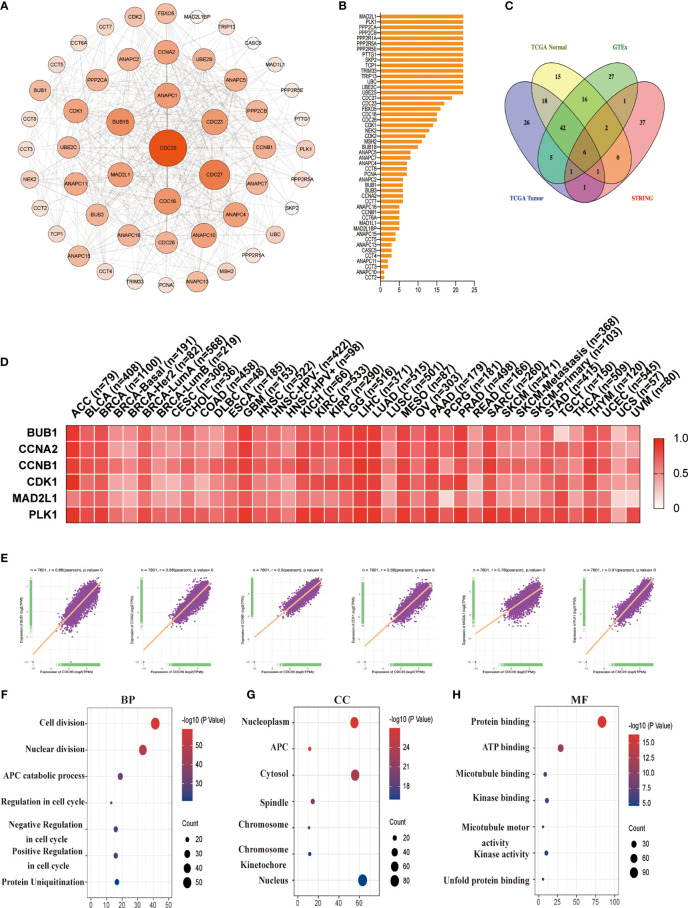
*CDC20*-related gene enrichment analysis. **(A)** The interaction network of Cdc20-binding proteins according to experimental evidence using the STRING tool is displayed. **(B)** The names and number of interaction nodes of the Cdc20-binding proteins according to experimental evidence using the STRING tool are displayed. **(C)** The Venn Diagram of the top 100 *CDC20*-correlated genes in TCGA tumor tissues, normal tissues, and GTEx database, as well as the gene encoding Cdc20-binding proteins according to experimental evidence using the STRING tool is displayed. **(D)** The corresponding heatmap of the intersection genes (*BUB1, CCNA2, CCNB1, CDK1, MAD2L1, PLK1*) in the detailed cancer types are displayed. **(E)** A pan-cancer correlation analysis of the *CDC20* mRNA levels and the top 6 correlated genes (*BUB1, CCNA2, CCNB1, CDK1, MAD2L1, PLK1*) was conducted. **(F–H)** Based on the Cdc20-binding and interacted genes, the dot plot for the molecular function data in GO analysis is performed.

Next, we aim to investigate the mutually molecular functions of *CDC20* in multiple tumor genesis at the pan-cancer level. To this end, we first reviewed the interaction network of Cdc20-binding proteins according to experimental evidence using the STRING tool and listed the top 50 genes in **Figure 6B** (**Figures 6A, B**). Secondly, to obtain a list of *CDC20* related genes, the GEPIA2 was used to analyze all tumor expression data of TCGA and obtained the top 100 genes that significantly correlated with CDC20 gene expression. The top 100 *CDC20*-correlated genes in TCGA tumor tissues, normal tissues, and GTEx database, as well as the gene encoding Cdc20-binding proteins according to experimental evidence using the STRING tool, were analyzed by the Venn diagram (**Figure 6C**). According to the Venn diagram, 6 genes were located in both the interaction group and the correlated list, including *BUB1, CCNA2, CCNB1, CDK1, MAD2L1* and *PLK1*. Then we profiled the tissue-wise expression of the 6 intersection genes in different cancer types using an interactive heatmap by GEPIA2 (**Figure 6D**). There was a positive correlation between *CDC20* and the above 6 genes in the majority of detailed cancer types. To be more specific, we performed a pairwise gene Pearson correlation analysis of *CDC20* and the 6 genes using the TCGA pan-cancer dataset with ggplot2 package. The expression levels of *BUB1, CCNA2, CCNB1, CDK1, MAD2L1*, and *PLK1* were significantly and positively correlated with *CDC20* (**Figure 6E**). To further analyze the pathways involved in the Cdc20-binding and interacted genes, the dot plot for the molecular function data in GO enrichment analysis was performed and indicated that most of these genes were linked to the pathways of the cell cycle, protein ubiquitination, kinase activity, and protein binding (**Figures 6F–H**). These results together show that the Cdc20 functions as an oncogenic protein in part through promoting its substrate poly-ubiquitination and binding with the related proteins.

### The Functional Validation of Cdc20

**Figure 7:**
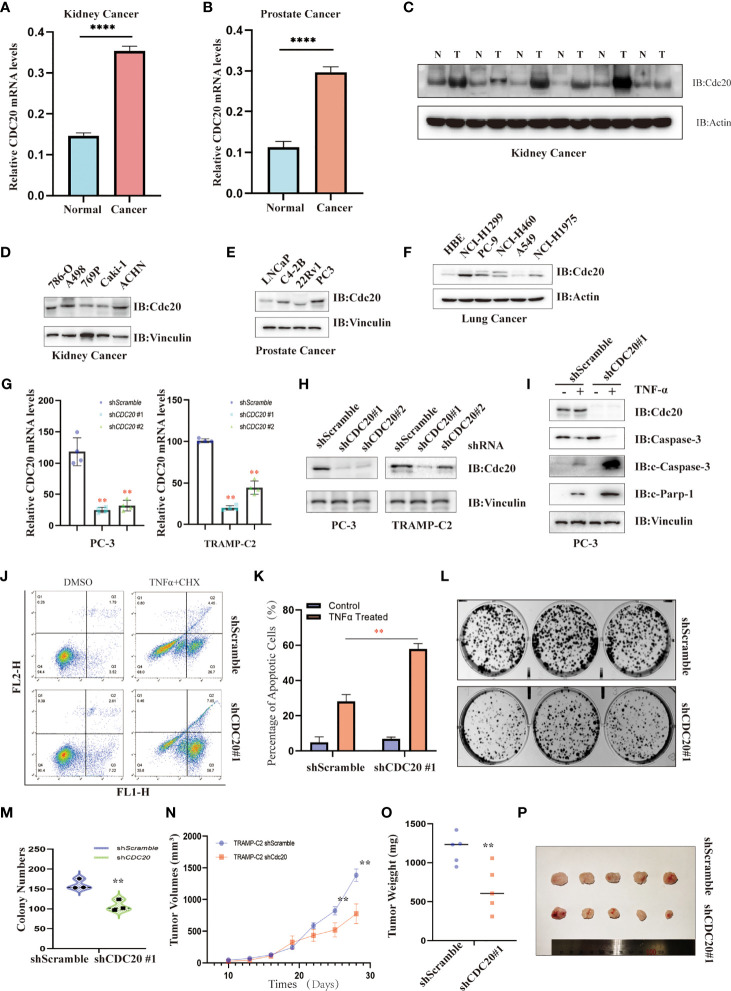
The functional validation of Cdc20. **(A, B)** Oncopression database was utilized to verify the differential expression of *CDC20* in the kidney **(A)** and prostate cancer **(B)**, as well as their matched normal tissues. **P < 0.01. **(C)** Immunoblotting was used to detect the abundance of Cdc20 levels in tumor tissues (T) and matched normal tissues (N) of renal cell carcinoma. **(D–F)** Immunoblotting was used to detect the abundance of Cdc20 levels in cancer cell lines derived from kidney cancer **(D)**, prostate cancer **(E)**, and lung cancer **(F)**. **(G)** Quantitative RT-PCR was utilized to detect the relative *CDC20* mRNA levels in PC-3 and TRAMP-C2 cell lines with or without *CDC20* stably knockdown, respectively. **(H)** Immunoblotting was used to detect the abundance of Cdc20 levels in PC-3 and TRAMP-C2 cell lines with or without *CDC20* stably knockdown, respectively. **(I)** Immunoblotting was used to detect the abundance of Cdc20 and apoptosis markers in PC-3 with or without *CDC20* stably knockdown treated with TNF-α (20 ng/ml) and Cycloheximide (10μM) for 4 hours before sampling. **(J, K)** Annexin-V and Propidium Iodide staining followed by FACS analysis to detect the proportion of apoptotic PC3 cells with or without *CDC20* stably knockdown treated with TNF-α (20 ng/ml) and Cycloheximide (10μM) for 4 hours before staining **(J)**. The quantification of three independent replicates was statistically analyzed and visualized **(K)**. **(L, M)** The representative photograph of colonic formation results in PC3 cells with or without *CDC20* stably knockdown **(L)**. The quantification of three independent replicates was statistically analyzed and visualized **(M)**. **(N–P)** A subcutaneous tumor mouse model was generated by injection of 5*10^5^ TRAMP-C2 cells with or without knockdown of *CDC20* in immune-competent C57BL/6J mice. The tumor volume **(N)** and weight **(O)** were recorded and statistically analyzed. Tumor xenografts of each group are shown **(P)**. **P < 0.01; ****P < 0.0001.

To further confirm the analysis from TCGA, GTEx, and CPTAC datasets, the Oncopression database was utilized to verify the differential expression of *CDC20* in cancer and their matched normal tissues. As shown in **Figures 7A, B** and **S3**, the expression difference of *CDC20* between tumors and matched control tissues were significantly increased in prostate cancer, kidney cancer, bladder cancer, breast cancer, colon cancer, esophageal cancer, gastric cancer, head and neck cancer, lung cancer, liver cancer, uterine cancer and pancreatic cancer, which was consistent with the previous findings in TCGA and GTEx dataset. Next, based on clinical samples from renal cell carcinoma and matched normal tissues, the abundance of Cdc20 protein was dramatically increased in tumor tissues (**Figure 7C**). In addition, we further compared the translational expression of Cdc20 across 15 familiar cell lines in cancer research to select the optimal model for the functional analysis of Cdc20 (**Figures 7D–F**). Immunoblotting showed that Cdc20 was highly expressed in 786-O, A498, and ACHN among cell lines in kidney cancer, and in PC3 of 4 prostate cancer cell lines, and NCI-H1299 and PC-9 of 6 lung cancer cell lines (Figures D-F). Then we generated stable cell lines with CDC20 knockdown in prostate cancer background with lentiviral shRNA virus. The knockdown effects were confirmed by qRT-PCR and immunoblotting in PC3 and TRAMP-C2 cell lines (**Figures 7G, H**). Next, we found that PC3 cells with *CDC20* knockdown became sensitive to TNF-α induced apoptosis (**Figures 7I–K**). Moreover, the knockdown of *CDC20* inhibits the clonic formation ability of prostate cancer cells (**Figures 7L, M**). As Cdc20 might induce immune suppression of microenviroment through the crosstalk with CAFs or MDSCs, we sought to explore the role of *CDC20* in immune-competent mice models. Here, we reported that knockdown of *CDC20* significantly inhibits the tumor growth in C57BL/6J mice, which was consistent with our previous findings in the immune infiltration analysis (**Figures 7N–P**). All the preliminary validation indicated that targeting Cdc20 could be a promising strategy and warrants further investigation.

## Discussion

Here we have identified *CDC20* plays a key role in the various human cancers. Our results showed that the expression levels of the *CDC20* gene were significantly elevated in prostate cancer, kidney cancer, bladder cancer, breast cancer, colon cancer, esophageal cancer, gastric cancer, head and neck cancer, lung cancer, liver cancer, uterine cancer, and pancreatic cancer. In addition, the expression of *CDC20* was significantly and positively correlated with the increase of clinical stages in multiple cancer types, including adrenal cancer, breast cancer, kidney cancer, and lung cancer. Among 33 cancer types in the TCGA dataset, the high expression of *CDC20* was correlated with poor prognosis in 10 cancer types, which further supports its oncogenic role in tumor progression. We also proved that the transcriptome data was consistent with the transcriptional abundance of Cdc20 by the CPTAC dataset, and the expression levels of phosphorylated Cdc20 in primary tumors were elevated and correlated with increased tumor grade. Next, we sought to elucidate the oncogenic role by analyzing its association with immune infiltration. For most cancer types, the *CDC20* expression was positively correlated with the infiltration of CAFs and MDSCs, while the correlation was significantly negative in a small group of cancer including BRCA. To understand its functional activity, we explored the classic Cdc20 downstream substrates, which were found to be mutually exclusive with the expression of Cdc20. In the end, the molecular function of Cdc20 in pan-cancer analysis indicated that *BUB1, CCNA2, CCNB1, CDK1, MAD2L1*, and *PLK1* might play a critical role in its oncogenic function. In addition, the expression and function of Cdc20 were further validated in our clinical samples and cancer cell lines, and immune-competent mice models. All these findings indicated that targeting Cdc20 could be a promising strategy and warrants further investigation. Previous studies have reported that CDC20, CDK1, CCNB1, and BUB1 may serve as potential biomarkers of pediatric adrenal carcinoma and as potential targets for therapeutic approaches (22). Our results presented here indicated that *CDC20* also plays a critical role in the progression of adrenal carcinoma. The expression of *CDC20* was significantly and positively correlated with the increase of clinical stages in adrenal carcinoma. In adrenal carcinoma, the *CDC20* expression was significantly associated with the poor OS and PFS, the HR of both OS and PFS were the highest among all cancer types in TCGA. Compared with prostate cancer, lung cancer, or breast cancer, adrenal carcinoma is rare and the experimental evidence is quite limited (23). In addition to adrenal carcinoma, it remains elusive how Cdc20 executes its oncogenic function in specific cancer. It is plausible that Cdc20 plays a central role and might be one of the driver factors in the tumorigenesis of adrenal carcinoma. Increasing evidence showed that Cdc20 exhibits an oncogenic function and targeting Cdc20 could be a novel strategy for combating human cancers (24, 25). Notably, our results showed that the high expression of *CDC20* was negatively correlated with OS in multiple cancer types, including ACC, KIRC, KIRP, LGG, LIHC, LUAD, MESO, and SKCM. Moreover, the phosphorylated Cdc20 could be more functional active in performing its oncogenic role in cancer. By analyzing the abundance of Cdc20 and its substrates, CDC20 is probably functionally active in the *CDC20* high-expression cancer types. Thus, the blockade of Cdc20 by small molecular inhibitors could be a plausible therapy in the indicated cancer types (26). Importantly, recent studies have reported a group of specific inhibitors of Cdc20 in the pre-clinical model of different kinds of cancer (27, 28). The tumor xenografts in immune-competent mice with or without *CDC20* knockdown were shown to have a dramatic shrinkage in tumor volume, compared with the knockdown of *CDC20* in the immune-deficient mice model as previous reproted (29). As *CDC20* expression correlated with CAFs and MDSCs, it is proposed that the anti-tumor effects of targeting Cdc20 could be augmented by the immune system. Our previous work has proved that targeting Cdc20 sensitized the docetaxel-resistant prostate cancer cells to chemotherapy (30). In keeping with that notion, as prostate cancer is androgen-dependent at the early stage, the combination of Cdc20 inhibitors with androgen inhibitors warrants further exploration (31). Based on current evidence, it is plausible that the combination therapy of Cdc20 with immune therapy could also be potentially beneficial for cancer patients with *CDC20* high expression

Therefore, our studies delineated the oncogenic role of *CDC20* and its prognostic significance at the pan-cancer level and proved its functional activity in Cdc20 high expression cancer types. As personalized targeted therapy utilizing individual tumor genetic status becomes more readily available in cancer diagnostics and treatment, we envision that our studies will provide the rationale for further exploration and developing novel therapeutic strategies in selected cancer types indicated in this study. Above all, these observation merits further molecular assays to understand the potential role of Cdc20 in tumorigenesis.

## Data Availability Statement

The datasets presented in this study can be found in online repositories. The names of the repository/repositories can be found here: http://cancergenome.nih.gov/.

## Ethics Statement

The studies involving human participants were reviewed and approved by the Institutional Review Boards of Shandong First Medical University. The patients/participants provided their written informed consent to participate in this study. The animal study was reviewed and approved by The Animal Care and Use Committee of Shandong First Medical University. Written informed consent was obtained from the individual(s) for the publication of any potentially identifiable images or data included in this article.

## Author Contributions

JL, QL, and FW provided the idea and design the study. YS, JC, and HL performed the data mining. KY and YL analyzed the data. FW and YS wrote the manuscript. All authors contributed to the article and approved the submitted version.

## Funding

This project was supported by Shandong Provincial Nature Science Foundation (ZR2020QH240), the Clinical Medicine Innovation Program of Jinan City (202019125), the National Nature Science Foundation of China (NSFC82002719), and the Shandong Provincial Key Research and Development Project (2019GSF107058 & 2019GSF108232).

## Conflict of Interest

The authors declare that the research was conducted in the absence of any commercial or financial relationships that could be construed as a potential conflict of interest.

## Publisher’s Note

All claims expressed in this article are solely those of the authors and do not necessarily represent those of their affiliated organizations, or those of the publisher, the editors and the reviewers. Any product that may be evaluated in this article, or claim that may be made by its manufacturer, is not guaranteed or endorsed by the publisher.

## Supplementary Material

Supplementary Figure 1 The mRNA expression of *CDC20* gene in different types of TCGA tumors. Box-plots of mRNA expression for ESCA **(A)**, HNSC **(B)**, LIHC **(C)**, LUSC **(D)**, PRAD **(E)**, READ **(F)**, STAD **(G)**, UCEC **(H)**, CHOL **(I)**, and PAAD **(J)**, with significantly elevated *CDC20* expression in malignant tissues according to the TCGA pan-cancer dataset. Besides, expression data of normal tissues from GTEx were included as normal controls. * P < 0.001.

Supplementary Figure 2 The correlation between *CDC20* expression and immune infiltration of myeloid-derived suppressor cells. **(A)** The potential correlation between the expression level of the *CDC20* gene and the infiltration level of myeloid-derived suppressor cells (MDSCs) across 33 subtypes of cancer in TCGA using different algorithms by TIMER2.0, visualized in a heatmap by Spearmans’ Rho value. **(B)** The expression levels of the *CDC20* gene are positively correlated with the infiltration levels of MDSCs by the cut-off value of 0.5 of Spearmans’ Rho Value in selected cancer types.

Supplementary Figure 3 The mRNA expression of *CDC20* gene in different types of tumors and matched normal tissues. The expression difference of *CDC20* between tumors and matched control tissues were significantly increased in bladder cancer **(A)**, breast cancer **(B)**, colon cancer **(C)**, esophageal cancer **(D)**, gastric cancer **(E)**, head and neck cancer **(F)**, lung cancer **(G)**, liver cancer **(H)**, uterine cancer **(I)** and pancreatic cancer **(J)**, based on the Oncopression dataset. *** P < 0.001.

Supplementary Table 1 The abbreviations of cancer types in the TCGA project.

## References

[1] Piano V Alex A Stege P Maffini S Stoppiello GA Huis PJ. CDC20 Assists Its Catalytic Incorporation in the Mitotic Checkpoint Complex. Science (2021) 371(6524):67–71. 10.1126/science.abc1152 33384373

[2] Schrock MS Stromberg BR Scarberry L Summers MK. APC/C Ubiquitin Ligase: Functions and Mechanisms in Tumorigenesis. Semin Cancer Biol (2020) 67(Pt 2):80–91. 10.1016/j.semcancer.2020.03.001 32165320 PMC7483777

[3] Yamano H. APC/C: Current Understanding and Future Perspectives. F1000Res (2019) 8:1–15. 10.12688/f1000research.18582.1 PMC6534075 31164978

[4] Wu F Dai X Gan W Wan L Li M Mitsiades N. Prostate Cancer-Associated Mutation in SPOP Impairs its Ability to Target Cdc20 for Poly-Ubiquitination and Degradation. Cancer Lett (2017) 385:207–14. 10.1016/j.canlet.2016.10.021 PMC5148662 27780719

[5] Zeng X Sigoillot F Gaur S Choi S Pfaff KL Oh DC. Pharmacologic Inhibition of the Anaphase-Promoting Complex Induces a Spindle Checkpoint-Dependent Mitotic Arrest in the Absence of Spindle Damage. Cancer Cell (2010) 18(4):382–95. 10.1016/j.ccr.2010.08.010 PMC2957475 20951947

[6] Wan L Tan M Yang J Inuzuka H Dai X Wu T. APC(Cdc20) Suppresses Apoptosis Through Targeting Bim for Ubiquitination and Destruction. Dev Cell (2014) 29(4):377–91. 10.1016/j.devcel.2014.04.022 PMC4081014 24871945

[7] Sackton KL Dimova N Zeng X Tian W Zhang M Sackton TB. Synergistic Blockade of Mitotic Exit by Two Chemical Inhibitors of the APC/C. Nature (2014) 514(7524):646–9. 10.1038/nature13660 PMC4214887 25156254

[8] Mao Y Li K Lu L Si-Tu J Lu M Gao X. Overexpression of Cdc20 in Clinically Localized Prostate Cancer: Relation to High Gleason Score and Biochemical Recurrence After Laparoscopic Radical Prostatectomy. Cancer Biomark (2016) 16(3):351–8. 10.3233/CBM-160573 PMC13016504 26889981

[9] Jiang J Jedinak A Sliva D. Ganodermanontriol (GDNT) Exerts its Effect on Growth and Invasiveness of Breast Cancer Cells Through the Down-Regulation of CDC20 and uPA. Biochem Biophys Res Commun (2011) 415(2):325–9. 10.1016/j.bbrc.2011.10.055 22033405

[10] Rajkumar T Sabitha K Vijayalakshmi N Shirley S Bose MV Gopal G. Identification and Validation of Genes Involved in Cervical Tumourigenesis. BMC Cancer (2011) 11:80. 10.1186/1471-2407-11-80 21338529 PMC3050856

[11] Marucci G Morandi L Magrini E Farnedi A Franceschi E Miglio R. Gene Expression Profiling in Glioblastoma and Immunohistochemical Evaluation of IGFBP-2 and CDC20. Virchows Arch (2008) 453(6):599–609. 10.1007/s00428-008-0685-7 18953566

[12] Ouellet V Guyot MC Le Page C Filali-Mouhim A Lussier C Tonin PN. Tissue Array Analysis of Expression Microarray Candidates Identifies Markers Associated With Tumor Grade and Outcome in Serous Epithelial Ovarian Cancer. Int J Cancer (2006) 119(3):599–607. 10.1002/ijc.21902 16572426

[13] Amador V Ge S Santamaria PG Guardavaccaro D Pagano M. APC/C(Cdc20) Controls the Ubiquitin-Mediated Degradation of P21 in Prometaphase. Mol Cell (2007) 27(3):462–73. 10.1016/j.molcel.2007.06.013 PMC2000825 17679094

[14] Lara-Gonzalez P Moyle P Budrewicz MW Mendoza-Lopez J Oegema K Desai A. The G2-To-M Transition Is Ensured by a Dual Mechanism That Protects Cyclin B From Degradation by Cdc20-Activated APC/C. Dev Cell (2019) 51(3):313–25.e10. 10.1016/j.devcel.2019.09.005 31588029 PMC7778526

[15] Harley ME Allan LA Sanderson HS Clarke PR. Phosphorylation of Mcl-1 by CDK1-Cyclin B1 Initiates its Cdc20-Dependent Destruction During Mitotic Arrest. EMBO J (2010) 29(14):2407–20. 10.1038/emboj.2010.112 PMC2910263 20526282

[16] Shi M Dai WQ Jia RR Zhang QH Wei J Wang YG. APC(CDC20)-Mediated Degradation of PHD3 Stabilizes HIF-1a and Promotes Tumorigenesis in Hepatocellular Carcinoma. Cancer Lett (2021) 496:144–55. 10.1016/j.canlet.2020.10.011 33039559

[17] Wang W Wu T Kirschner MW. The Master Cell Cycle Regulator APC-Cdc20 Regulates Ciliary Length and Disassembly of the Primary Cilium. Elife (2014) 3:e03083. 10.7554/eLife.03083 25139956 PMC4135350

[18] Lara-Gonzalez P Kim T Oegema K Corbett K Desai A. A Tripartite Mechanism Catalyzes Mad2-Cdc20 Assembly at Unattached Kinetochores. Science (2021) 371(6524):64–7. 10.1126/science.abc1424 PMC8191211 33384372

[19] Chen F Chandrashekar DS Varambally S Creighton CJ. Pan-Cancer Molecular Subtypes Revealed by Mass-Spectrometry-Based Proteomic Characterization of More Than 500 Human Cancers. Nat Commun (2019) 10(1):5679. 10.1038/s41467-019-13528-0 31831737 PMC6908580

[20] Xiong C Wang Z Wang G Zhang C Jin S Jiang G. Identification of CDC20 as an Immune Infiltration-Correlated Prognostic Biomarker in Hepatocellular Carcinoma. Invest New Drugs (2021). 10.1007/s10637-021-01126-1 33942202

[21] Li T Fu J Zeng Z Cohen D Li J Chen Q. TIMER2.0 for Analysis of Tumor-Infiltrating Immune Cells. Nucleic Acids Res (2020) 48(W1):W509–14. 10.1093/nar/gkaa407 PMC7319575 32442275

[22] Kulshrestha A Suman S Ranjan R. Network Analysis Reveals Potential Markers for Pediatric Adrenocortical Carcinoma. Onco Targets Ther (2016) 9:4569–81. 10.2147/OTT.S108485 PMC4968868 27555782

[23] Francis JC Gardiner JR Renaud Y Chauhan R Weinstein Y Gomez-Sanchez C. HOX Genes Promote Cell Proliferation and Are Potential Therapeutic Targets in Adrenocortical Tumours. Br J Cancer (2021) 124(4):805–16. 10.1038/s41416-020-01166-z PMC7884796 33214683

[24] Kidokoro T Tanikawa C Furukawa Y Katagiri T Nakamura Y Matsuda K. CDC20, A Potential Cancer Therapeutic Target, Is Negatively Regulated by P53. Oncogene (2008) 27(11):1562–71. 10.1038/sj.onc.1210799 17873905

[25] Wang L Zhang J Wan L Zhou X Wang Z Wei W. Targeting Cdc20 as a Novel Cancer Therapeutic Strategy. Pharmacol Ther (2015) 151:141–51. 10.1016/j.pharmthera.2015.04.002 PMC4457591 25850036

[26] Richeson KV Bodrug T Sackton KL Yamaguchi M Paulo JA Gygi SP. Paradoxical Mitotic Exit Induced by a Small Molecule Inhibitor of APC/C(Cdc20). Nat Chem Biol (2020) 16(5):546–55. 10.1038/s41589-020-0495-z PMC7289404 32152539

[27] Cheng S Castillo V Sliva D. CDC20 Associated With Cancer Metastasis and Novel Mushroom Derived CDC20 Inhibitors With Antimetastatic Activity. Int J Oncol (2019) 54(6):2250–6. 10.3892/ijo.2019.4791 31081056

[28] Gao Y Zhang B Wang Y Shang G. Cdc20 Inhibitor Apcin Inhibits the Growth and Invasion of Osteosarcoma Cells. Oncol Rep (2018) 40(2):841–8. 10.3892/or.2018.6467 29901174

[29] Gao Y Wen P Chen B Hu G Wu L Xu A. Downregulation of CDC20 Increases Radiosensitivity Through Mcl-1/P-Chk1-Mediated DNA Damage and Apoptosis in Tumor Cells. Int J Mol Sci (2020) 21(18):6692. 10.3390/ijms21186692 PMC7555290 32932732

[30] Wu F Lin Y Cui P Li H Zhang L Sun Z. Cdc20/p55 Mediates the Resistance to Docetaxel in Castration-Resistant Prostate Cancer in a Bim-Dependent Manner. Cancer Chemother Pharmacol (2018) 81(6):999–1006. 10.1007/s00280-018-3578-8 29605876

[31] Giatromanolaki A Fasoulaki V Kalamida D Mitrakas A Kakouratos C Lialiaris T. CYP17A1 and Androgen-Receptor Expression in Prostate Carcinoma Tissues and Cancer Cell Lines. Curr Urol (2019) 13(3):157–65. 10.1159/000499276 PMC6944932 31933595

