# Supplementary figures and images for: The Oncogenic Role of APC/C Activator Protein Cdc20 by an Integrated Pan-Cancer Analysis in Human Tumors
Source: Front Oncol. 2021 Aug 30;11:721797. doi: 10.3389/fonc.2021.721797 (PMC8435897; doi:10.3389/fonc.2021.721797)

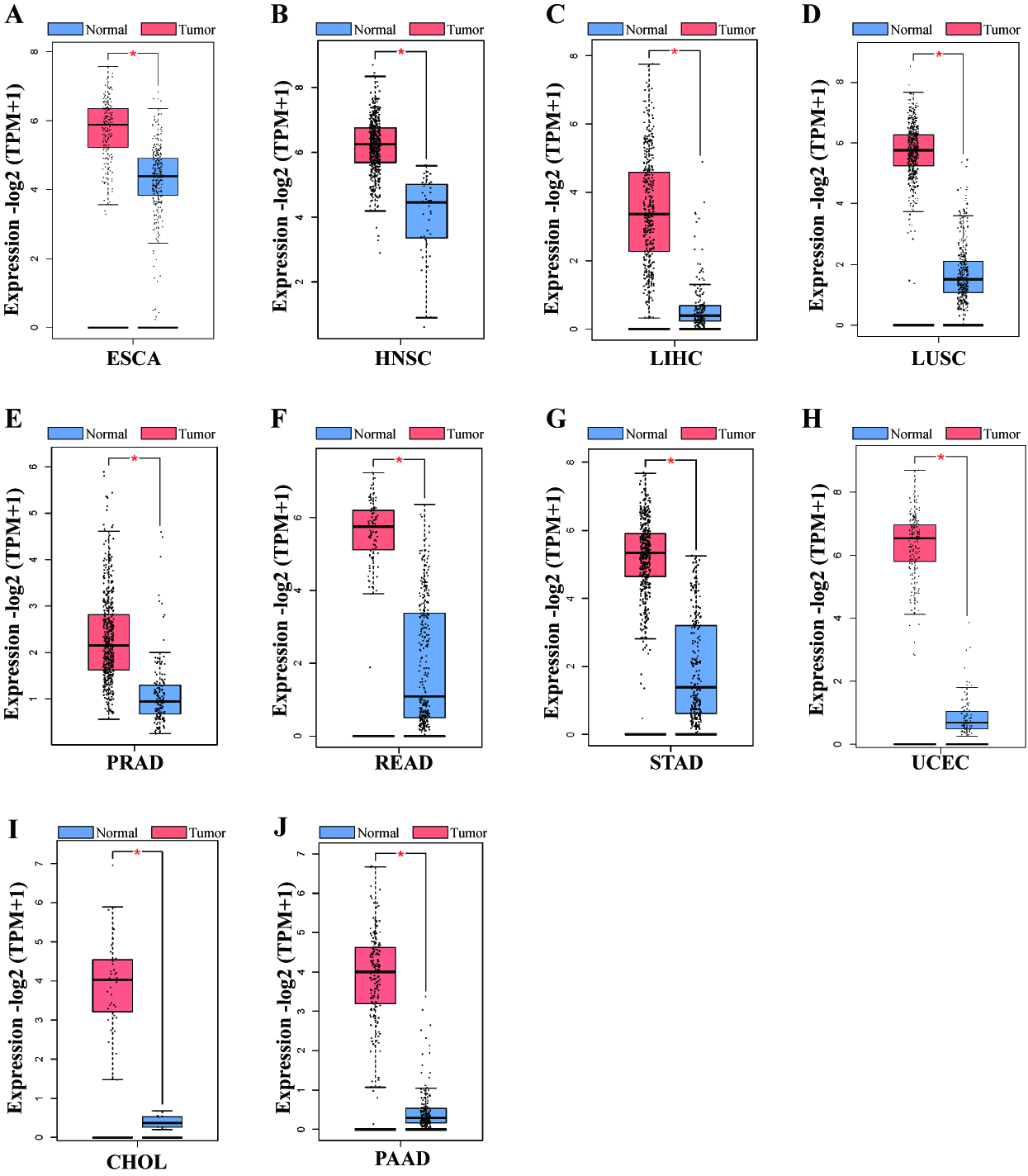

Supplement: Supplementary Figure 1 — The mRNA expression of CDC20 gene in different types of TCGA tumors. Box-plots of mRNA expression for ESCA (A), HNSC (B), LIHC (C), LUSC (D), PRAD (E), READ (F), STAD (G), UCEC (H), CHOL (I), and PAAD (J), with significantly elevated CDC20 expression in malignant tissues according to the TCGA pan-cancer dataset. Besides, expression data of normal tissues from GTEx were included as normal controls. * P < 0.001. [file Image_1.tif]

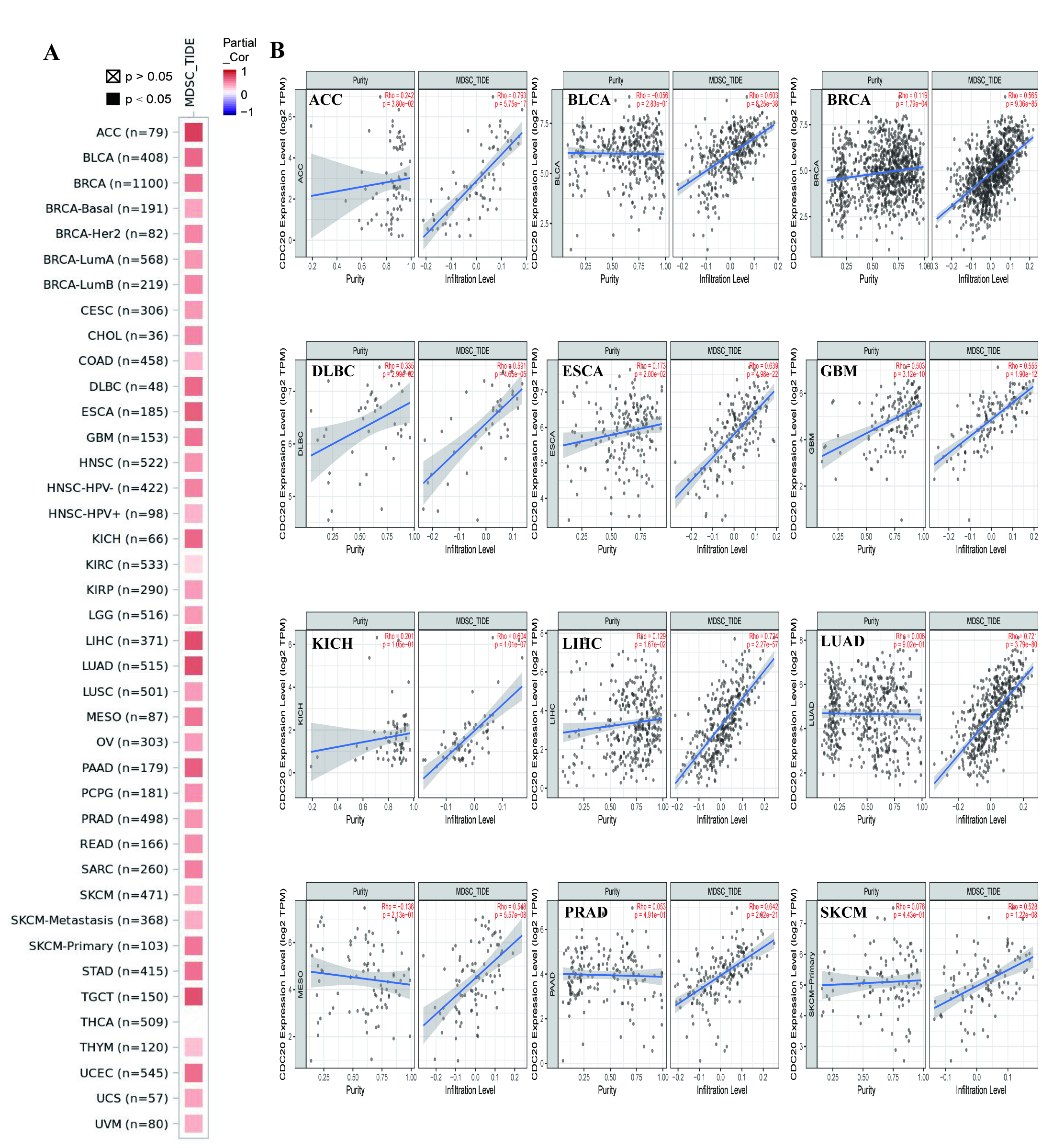

Supplement: Supplementary Figure 2 — The correlation between CDC20 expression and immune infiltration of myeloid-derived suppressor cells. (A) The potential correlation between the expression level of the CDC20 gene and the infiltration level of myeloid-derived suppressor cells (MDSCs) across 33 subtypes of cancer in TCGA using different algorithms by TIMER2.0, visualized in a heatmap by Spearmans’ Rho value. (B) The expression levels of the CDC20 gene are positively correlated with the infiltration levels of MDSCs by the cut-off value of 0.5 of Spearmans’ Rho Value in selected cancer types. [file Image_2.tif]

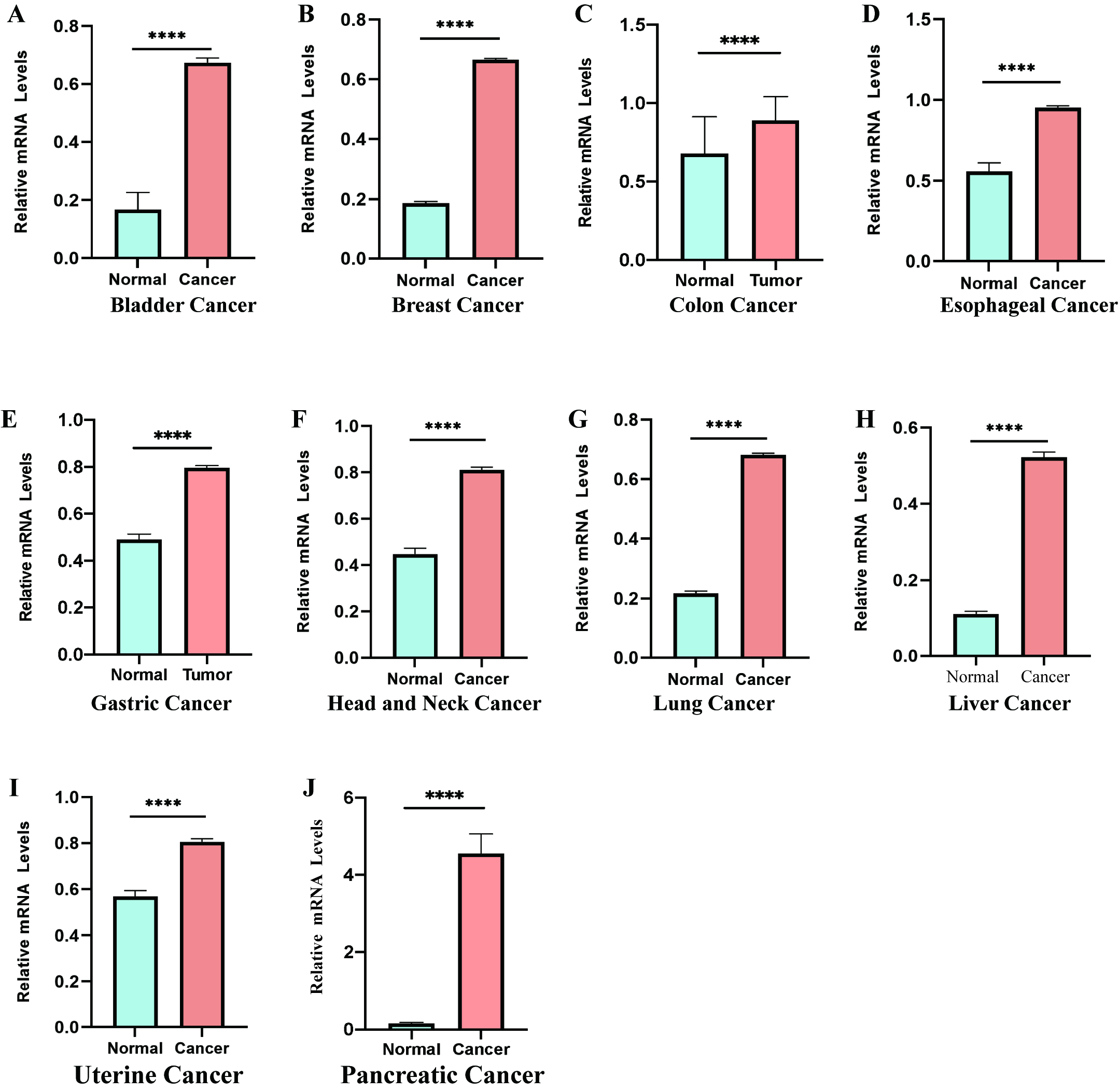

Supplement: Supplementary Figure 3 — The mRNA expression of CDC20 gene in different types of tumors and matched normal tissues. The expression difference of CDC20 between tumors and matched control tissues were significantly increased in bladder cancer (A), breast cancer (B), colon cancer (C), esophageal cancer (D), gastric cancer (E), head and neck cancer (F), lung cancer (G), liver cancer (H), uterine cancer (I) and pancreatic cancer (J), based on the Oncopression dataset. *** P < 0.001. [file Image_3.tif]
